# Study protocol for HGCSG1801: A multicenter, prospective, phase II trial of second-line FOLFIRI plus aflibercept in patients with metastatic colorectal cancer refractory to anti-EGFR antibodies

**DOI:** 10.3389/fonc.2022.939425

**Published:** 2022-11-09

**Authors:** Hiroshi Nakatsumi, Yoshito Komatsu, Tetsuhito Muranaka, Satoshi Yuki, Yasuyuki Kawamoto, Kazuaki Harada, Masayoshi Dazai, Miki Tateyama, Yusuke Sasaki, Takuto Miyagishima, Yasushi Tsuji, Masaki Katagiri, Michio Nakamura, Susumu Sogabe, Kazuteru Hatanaka, Takashi Meguro, Tomoe Kobayashi, Atsushi Ishiguro, Osamu Muto, Yoshiaki Shindo, Masahito Kotaka, Takayuki Ando, Ryo Takagi, Naoya Sakamoto, Yu Sakata

**Affiliations:** ^1^ Department of Gastroenterology, National Hospital Organization Hokkaido Medical Center, Sapporo, Japan; ^2^ Division of Cancer Center, Hokkaido University Hospital, Sapporo, Japan; ^3^ Department of Internal Medicine, Wakkanai City Hospital, Wakkanai, Japan; ^4^ Department of Gastroenterology and Hepatology, Hokkaido University Hospital, Sapporo, Japan; ^5^ Department of Gastroenterology, Sapporo Medical Center NTT EC, Sapporo, Japan; ^6^ Department of Internal Medicine, Tomakomai Nissho Hospital, Tomakomai, Japan; ^7^ Department of Medical Oncology, Hakodate Central General Hospital, Hakodate, Japan; ^8^ Department of Medical Oncology, Kushiro Rosai Hospital, Kushiro, Japan; ^9^ Department of Medical Oncology, Tonan Hospital, Sapporo, Japan; ^10^ Department of Gastroenterology, Sapporo Hokuyu Hospital, Sapporo, Japan; ^11^ Department of Gastroenterology, Sapporo City General Hospital, Sapporo, Japan; ^12^ Department of Medical Oncology, KKR Sapporo Medical Center, Sapporo, Japan; ^13^ Department of Gastroenterology, Hakodate Municipal Hospital, Hakodate, Japan; ^14^ Department of Internal Medicine, Hokkaido Gastroenterology Hospital, Sapporo, Japan; ^15^ Department of Gastroenterology, Tomakomai City Hospital, Tomakomai, Japan; ^16^ Department of Medical Oncology, Teine Keijinkai Hospital, Sapporo, Japan; ^17^ Department of Medical Oncology, Japanese Red Cross Akita Hospital, Akita, Japan; ^18^ Department of Gastroenterological Surgery, Nakadori General Hospital, Akita, Japan; ^19^ General Cancer Center, Sano Hospital, Kobe, Japan; ^20^ The third department of Internal Medicine, University of Toyama, Toyama, Japan; ^21^ Clinical Research and Medical Innovation Center, Hokkaido University Hospital, Sapporo, Japan; ^22^ CEO, Misawa Municipal Misawa Hospital, Misawa, Japan

**Keywords:** colorectal cancer, chemotherapy, FOLFIRI, aflibercept, anti-EGFR antibodies

## Abstract

**Background:**

The first-line chemotherapy for patients with *RAS* and *BRAF* wild-type metastatic colorectal cancer (mCRC) commonly involves cytotoxic regimens, such as FOLFOX and FOLFIRI, combined with epidermal growth factor receptor (EGFR) antibodies. When progression occurs following anti-EGFR antibody-combined chemotherapy, anti-angiogenic inhibitors can be used as second-line treatment. Although randomized controlled trials have shown that anti-angiogenic inhibitors [bevacizumab, ramucirumab, and aflibercept (AFL)] carry survival benefit when combined with FOLFIRI as second-line chemotherapy, such trials did not provide data on patients with mCRC refractory to anti-EGFR antibody-combined chemotherapy. Therefore, our group planned a multicenter, nonrandomized, single-arm, prospective, phase II study to investigate the safety and efficacy of FOLFIRI plus AFL as a second-line chemotherapy for patients with mCRC refractory to oxaliplatin-based chemotherapy combined with anti-EGFR antibodies.

**Methods:**

FOLFIRI (irinotecan 180 mg/m^2^, *l*-leucovorin 200 mg/m^2^, bolus 5-FU 400 mg/m^2^, and infusional 5-FU 2400 mg/m^2^/46 h) and AFL (4 mg/kg) will be administered every 2 weeks until progression or unacceptable toxicities occur. The primary endpoint will be the 6-month progression-free survival (PFS) rate, whereas the secondary endpoints will include overall survival, PFS, response rate, disease control rate, adverse events, and relative dose intensity for each drug. A sample size of 41 participants will be required. This study will be sponsored by the Non-Profit Organization Hokkaido Gastrointestinal Cancer Study Group and will be supported by a grant from Sanofi.

**Discussion:**

There is only an observational study reporting data on FOLFIRI plus AFL for patients with mCRC who previously received anti-EGFR antibodies; therefore, a prospective clinical trial is needed. This study will prospectively evaluate the efficacy and safety of FOLFIRI plus AFL in patients with mCRC who are resistant to anti-EGFR antibodies and have limited data. Moreover, this study will reveal predictive biomarkers for AFL-based chemotherapy.

**Clinical trial registration:**

Japan Registry of Clinical Trials, jRCTs011190006. Registered 19 November, 2019, https://jrct.niph.go.jp/latest-detail/jRCTs011190006.

## Introduction

The incidence of colorectal cancer (CRC) has increased over the decades and is the second most common cause of cancer-related death in Japan. Estimates in 2016 revealed 201,446 new CRC cases, with 50,099 deaths throughout Japan (1). Metastatic CRC (mCRC) carries a poor prognosis with no currently available cure. Chemotherapy remains the standard treatment for mCRC and aims to prolong survival and improve patients’ quality of life. Nowadays, chemotherapy regimens, such as combinations of 5-fluorouracil (5-FU), leucovorin (LV), and oxaliplatin (FOLFOX) and 5-FU, LV, and irinotecan (FOLFIRI), have been recommended as first-line therapies usually in combination with monoclonal antibodies, such as an anti-vascular endothelial growth factor (VEGF)-A antibody [bevacizumab (BEV)] or anti-epidermal growth factor receptor (EGFR) antibodies (cetuximab or panitumumab) (2). Other studies have also recommended FOLFOXIRI, a combination chemotherapy comprising 5-FU, LV, oxaliplatin, and irinotecan (IRI), with or without BEV (3, 4). Chemotherapies comprising anti-EGFR antibodies have been recommended as the first-line chemotherapy for patients with *RAS* and *BRAF* wild-type mCRC. Indeed, a subgroup analysis of the FIRE-3 study showed that anti-EGFR antibody-based regimens promoted better survival outcomes than BEV-based regimens for left-sided mCRC (5), with several subgroup analyses of prospective clinical trials showing similar outcomes (6, 7). Nowadays, chemotherapy with anti-EGFR antibodies (cetuximab or panitumumab) has been the most recommended regimen in the ESMO-Asian consensus guidelines for patients with *RAS* wild-type left-sided mCRC (8).

Aflibercept (AFL) is a recombinant fusion protein containing portions of the extracellular domains of VEGF receptors 1 and 2 fused to the Fc portion of human immunoglobulin G1 (9). Interestingly, the VELOUR study, which compared FOLFIRI with or without AFL, showed that FOLFIRI plus AFL had better survival outcomes than FOLFIRI alone (median overall survival [OS]: 13.50 vs. 12.06 months, hazard ratio [HR] = 0.817, p = 0.0032) (9). Moreover, ML18147 (10) and RAISE (11) have found that BEV-based chemotherapy and FOLFIRI plus ramucirumab (RAM), a monoclonal antibody targeting VEGF receptor 2, were efficacious second-line therapies. ML18147 showed that continuous BEV plus second-line chemotherapy after progression with BEV plus first-line chemotherapy prolonged survival compared with second-line chemotherapy alone (median overall survival [OS]: 11.2 vs. 9.8 months, HR = 0.81, p = 0.0062) (10). RAISE also showed the efficacy of FOLFIRI plus RAM for patients with mCRC refractory to the first-line chemotherapy with BEV, oxaliplatin, and fluoropyrimidines compared with FOLFIRI plus placebo (median OS: 13.3 vs. 11.7 months, HR = 0.844, p = 0.0219) (11). Notably, ML18147 and RAISE have included participants who received BEV as their first-line chemotherapy, whereas VELOUR included those who received first-line chemotherapy without BEV. In the VELOUR study, the subgroup analysis defined by *RAS* or *BRAF* status showed that WT (all *RAS* wild-type and *BRAF* wild-type) and *BRAF* mutant-type (mut) tended to have better OS outcomes with FOLFIRI plus AFL (WT: HR = 0.76, 95% confidence interval [CI] 0.53–1.10; BRAF mut: HR = 0.42, 95% CI 0.16–1.09), which is a promising result for patients receiving first-line chemotherapy with anti-EGFR antibodies (12). However, the VELOUR study did not report results for patients who received first-line therapy with anti-EGFR antibodies, whereas little evidence regarding this population was presented. Therefore, no clinical studies have yet demonstrated the efficacy of adding a molecular-targeted agent to second-line chemotherapies for mCRC refractory to first-line treatment including anti-EGFR antibodies.

Considering the lack of evidence, second-line regimens have been selected based on physicians’ discretion or patient preference. Therefore, this multicenter clinical study sought to investigate the efficacy and safety of FOLFIRI plus AFL for unresectable advanced or recurrent CRC refractory to combination therapy with oxaliplatin and panitumumab or cetuximab.

## Materials and methods

### Study design and treatment

**Figure 1:**
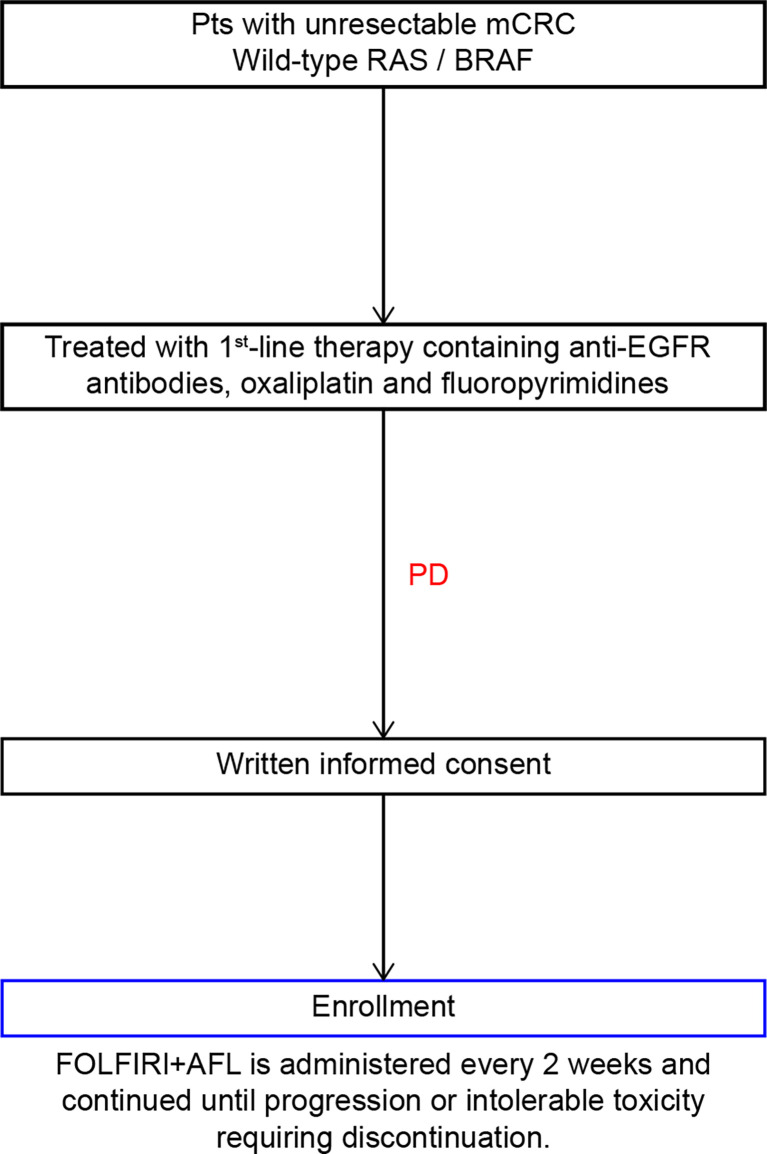
Study design. ECOG PS, Eastern Cooperative Oncology Group Performance Status.

This multicenter, nonrandomized, single-arm, prospective, phase II study was designed to investigate the safety and efficacy of FOLFIRI plus AFL as the second-line chemotherapy for patients with mCRC refractory to oxaliplatin-based chemotherapy combined with anti-EGFR antibodies (**Figure 1**). The inclusion and exclusion criteria are summarized in **Table 1**.

**Table 1:** Eligibility criteria.

Inclusion criteria
1. Age 20–85 years
2. Histologically confirmed colorectal adenocarcinoma
3. ECOG PS 0 or 1
4. Documented disease progression 90 days or more after the start of first-line chemotherapy containing anti-EGFR antibodies, oxaliplatin, and fluoropyrimidines
5. Adequate major organ and hematologic functions 14 days before registration
Neutrophil ≥ 1500/mm^3^
Platelet ≥ 100,000/mm^3^
Hemoglobin ≥ 9.0 g/dL
AST ≤ 100 U/L (pts with liver metastases: ≤ 200 U/L)
ALT ≤ 100 U/L (pts with liver metastases: ≤ 200 U/L)
Total bilirubin ≤ 2.0 mg/dL
Creatinine ≤ 1.5× upper limit of normal
Urine protein (UP) ≤ 1+ or UP/creatinine ratio (UPCR) <2.0 without hematuria (pts with UP ≥2+ and UPCR > 1; UP ≤3.5 g in 24-h urine collection without hematuria)
6. Expected survival time ≥6 months upon registration
7. With documented informed consent
**Exclusion criteria**
1. Symptomatic CNS invasion and/or brain metastases
2. Uncontrollable infections
3. Uncontrollable diarrhea, anorexia, or nausea
4. Uncontrollable hypertension
5. Uncontrollable, symptomatic plural effusion, or ascites
6. Inability of oral intake
7. Radiation to all lesions
8. UGT1A1 *6 homo, *28 homo, or *6 and *28 double hetero
9. History of other malignancies within 5 years (except for curative resection of carcinoma in situ)
10. Pregnant women
11. Irinotecan-containing regimen as first-line therapy
12. Following status:
Gastrointestinal or abdominal inflammations
Gastrointestinal or other bleeding
Hemorrhagic diathesis, coagulopathy, or treated with anticoagulants
Thromboembolism
No wound healing from major surgery
13. Administration of aflibercept-containing medications
14. Dihydropyrimidine dehydrogenase deficiency
15. Interstitial pneumonia or pulmonary fibrosis
16. Participation in the clinical trial is determined as unsuitable

ALT, alanine aminotransferase; AST, aspartate aminotransferase; CNS, central nervous system; ECOG PS, Eastern Cooperative Oncology Group Performance Status; EGFR, endothelial growth factor receptor; UGT1A1, uridine diphosphate glucuronosyltransferase 1A1.

**Figure 2:**
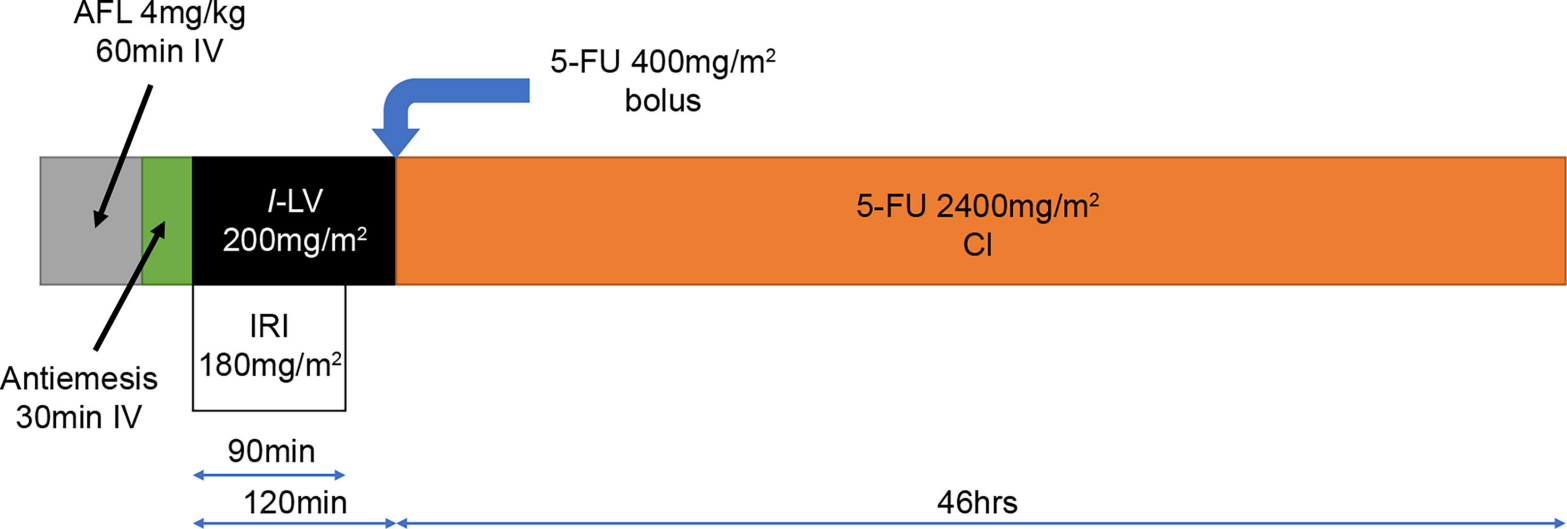
Treatment schedule for FOLFIRI+AFL. 5-FU, 5-fluorouracil; AFL, aflibercept beta; CI, continuous infusion; IRI, irinotecan; *l-*LV, *l-*leucovorin.

The treatment protocol starts 7 days after study registration; the treatment schedule is outlined in **Figure 2**. AFL (4 mg/kg) will be given as a continuous intravenous infusion over 60 min on day 1, followed by a continuous infusion of IRI (180 mg/m^2^) over 90 min and *l*-leucovorin (200 mg/m^2^) over 120 min. Immediately after completing the *l*-leucovorin infusion, 5-FU (400 mg/m^2^) will be provided *via* rapid intravenous injection, followed by a continuous intravenous infusion of 5-FU (2400 mg/m^2^) delivered over 46 h using an infusion pump. This 2-week treatment course will be repeated until disease progression or unacceptable toxicities occur.

This study will be conducted in accordance with the Declaration of Helsinki and Ethical Guidelines for Medical and Health Research Involving Human Subjects. HGCSG1801 received approval on October 9, 2019, by Hokkaido University Certified Review Board. All patients will be required to provide written informed consent before enrollment.

### Endpoints and assessments

The primary endpoint will be the 6-month progression-free survival (PFS) rate, whereas the secondary endpoints will include OS, PFS, overall response rate (ORR), disease control rate (DCR), safety profile, and relative dose intensity.

The OS period will be defined as the duration from the first administration of the study treatment until death due to any cause. Surviving cases will be censored on the last confirmed survival day. The PFS period will be defined as the duration from the first administration of the study treatment until disease progression or death due to any cause, whichever comes first. Cases with no confirmed progression will be censored on the last confirmed PFS date. ORR and DCR will be evaluated based on the Response Evaluation Criteria in Solid Tumors version 1.1 (13). Regarding safety, the frequency of adverse events will be evaluated using the Common Terminology Criteria for Adverse Events v5.0.

The exploratory endpoint will indicate the relationship between primary/secondary endpoints and changes in the following biomarkers: placental growth factor (PlGF), hepatocyte growth factor (HGF), interleukin (IL)-6, IL-8, angiopoietin-2, neuropillin-1, tissue inhibitor of metalloproteinase-1 (TIMP-1), soluble intercellular adhesion molecule-1, soluble vascular cell adhesion molecule-1, thrombospondin-2 (TSP-2), osteopontin (OPN), sVEGFR1, sVEGFR2, sVEGFR3, VEGF-A, VEGF-D, interferon gamma, and transforming growth factor β1.

### Biomarker analysis

Blood plasma will be collected three times (before study treatment initiation, before the fifth–sixth cycle, and after study discontinuation) to measure angiogenic factors mentioned earlier. These factors will be analyzed concurrently using the multiplex assay kit Ukit (G&G Science Co., Ltd.) from the MILLIPLEX^®^ MAP multiplex assay kit lineup by Millipore (www.merckmillipore.jp/1milliplex-jp).

The MILLIPLEX^®^ kit is a dedicated reagent for multiplex assay systems using Luminex xMAP^®^ technology (https://www.merckmillipore.com/JP/en/life-science-research/protein-detection-quantification/Immunoassay-Platform-Solutions/luminex-instruments/xmap-technology/OUGb.qB.D_kAAAFB6sYRRk_Q,nav). Samples will be incubated with magnetic bead analytes (MILLIPLEX^®^ MAP) stained stepwise with two fluorescent dyes to specifically bind the target protein to the magnetic bead analyte. Thereafter, the sample will be reacted with a biotin-labeled detection sample and fluorescently labeled with streptavidin-PE, followed by the identification of the magnetic bead analyte and fluorescence intensity quantification using two types of lasers. The analyses of these factors will be performed by GG.

### Statistical analysis

The full analysis set (FAS) will include all enrolled patients. However, patients with serious protocol violations (no informed consent or serious violations of the study procedures) will be excluded. The safety analysis set (SAS) will include all enrolled patients who receive one dose of the study treatment. All efficacy analyses will be performed in the FAS population, whereas safety analysis will be performed in the SAS population.

The VELOUR study reported 6-month PFS rates of 38.9% and 58.4% for FOLFIRI and FOLFIRI + AFL, respectively, as second-line chemotherapy for mCRC refractory to first-line chemotherapy containing oxaliplatin and fluoropyrimidines, respectively (9). Based on the aforementioned results, we hypothesized a 6-month PFS rate of 58.4% for FOLFIRI plus AFL in patients previously treated and exhibited progression with anti-EGFR agents plus oxaliplatin-based first-line chemotherapy. Given the threshold and expected 6-month PFS rates of 38.9% and 58.4%, respectively, as well as a two-sided significance level (alpha error) of 0.1, a registration period of 2 years, and a follow-up period of 18 months, 37 cases will be required when the probability that the lower limit of the CI calculated using the Greenwood formula (14) exceeds the threshold is 80%. Consequently, the target number of cases will be set at 41 to account for ineligible cases.

This study primarily sought to evaluate the efficacy of FOLFIRI + AFL. The main analysis will utilize the Kaplan–Meier method (15) to evaluate the 6-month PFS rate (i.e., primary endpoint) in the FAS population. The 90% CI will be calculated using the Greenwood formula for the observed 6-month PFS rate, with significance being indicated when the lower limit of the 90% CI exceeds the threshold of 38.9%.

Secondary endpoints will be analyzed to supplement the main analysis results. OS and PFS will be evaluated using the Kaplan–Meier method, and the ORR and DCR will be calculated using the Clopper–Pearson 95% CI (16).

## Discussion

Limited data have been available on the optimal second-line regimen for mCRC refractory to anti-EGFR antibodies. The QoLiTrap study reported promising data of FOLFIRI plus AFL for patients who previously received anti-EGFR antibodies (ORR 23.7%, median PFS 9.4 months and median OS 17.4 months); however, it is an observational study and included patients receiving FOLFIRI plus AFL as second- or later-line therapy (17). Therefore, a prospective clinical trial is needed.

The present study will evaluate the efficacy and safety of FOLFIRI plus AFL in patients who have progressed with EGFR inhibitors, as well as changes in angiogenesis-related biomarkers during FOLFIRI plus AFL chemotherapy, to identify predictive biomarkers for AFL-based chemotherapy.

Although no predictive markers for the efficacy of angiogenesis inhibitors, such as *RAS* or *BRAF*, in EGFR antibody therapy have previously been identified, some promising factors have been reported. Accordingly, PlGF and HGF were significantly increased during BEV-combined chemotherapy before imaging indicated progression, whereas VEGFR2 was significantly increased after BEV administration and then decreased at the time of progression (18). Moreover, patients with high IL-8 levels upon treatment initiation had a significantly shorter PFS versus those with low levels. A biomarker study in the CALGB80405 trial showed that low VEGF-D and low PlGF may indicate BEV effectiveness and that factors, including angiopoietin-2, HGF, ICAM-1, IL-6, OPN, TIMP-1, TSP-2, VCAM-1, VEGFR3, can be prognostic indicators (19).

The biomarker analysis in the VELOUR study (20, 21) revealed that high levels of VEGF-A, VEGFR2, PlGF, and VEGFR3 and low levels of IL-8 indicated the efficacy of FOLFIRI + AFL, with neuropilin-1 and angiopoietin-2 having been identified as prognostic factors.

The Angiogenesis Panel that will be used in the biomarker analysis of the present study is a kit that can simultaneously measure 17 angiogenic factors. By evaluating changes in these factors before, during, and after study treatment administration, prognostic and prognostic factors can be identified.

The results of this study will elucidate the efficacy and safety of FOLFIRI plus AFL in mCRC refractory to anti-EGFR antibodies, as well as identify biomarkers for such a regimen.

## Data availability statement

The original contributions presented in the study are included in the article/supplementary material. Further inquiries can be directed to the corresponding author.

## Ethics statement

HGCSG1801 received approval on 9/Oct/2019 by Hokkaido University Certified Review Board. The patients/participants provided their written informed consent to participate in this study.

## Author contributions

YoK, the chief investigator, designed and prepared the study protocol together with HN, TeM, and YaK. HN and TeM designed the biological specimen collection. HN, YoK, SY, YaK, KHar, MD, MT, YuSas, TMi, YT, MKa, MN, SS, KHat, TMe, TK, AI, OM, YoS, MKo, and TA will recruit and follow-up patients. RT is the chief statistician. NS and YuSaka will be coordinating the study. All authors contributed to the article and approved the submitted version.

## Funding

This study is sponsored by the Non-Profit Organization Hokkaido Gastrointestinal Cancer Study Group and is supported by a grant from Sanofi (Grant no. SGZ-2017-11875).

## Acknowledgments

We are grateful to all patients and investigators for their cooperation in the HGCSG1801 study. The authors would like to thank Enago (www.enago.jp) for the English language review.

## Conflict of interest

HN has received honoraria from Takeda, Sanofi, and Taiho; YoK has received honoraria from Taiho, Yakult Honsha, Takeda, Pfizer, Daiichi Sankyo, Sanofi, and Nipro and has received research funding from Taiho, Yakult Honsha, Daiichi Sankyo, and Sanofi; SY has received honoraria from Takeda, Sanofi, Taiho, and Yakult Honsha; YaK has received honoraria from Takeda, Kyowa Kirin; YT has received honoraria from Taiho, Takeda, and Sawai; MN has received honoraria from Daiichi Sankyo, Taiho, Kyowa Kirin, Takeda and Mochida and has received research funding from Takeda; SS has received honoraria from Sanofi and Daiichi Sankyo; YoS has received honoraria from Yakult Honsha; MKo has received honoraria from Yakult Honsha, Takeda, Sanofi, and Taiho; TA has received honoraria from Takeda and Daiichi Sankyo; NS has received research funding from Takeda; YuSaka has received honoraria from Yakult Honsha and Taiho.

The remaining authors declare that the research was conducted in the absence of any commercial or financial relationships that could be construed as a potential conflict of interest.

## Publisher’s note

All claims expressed in this article are solely those of the authors and do not necessarily represent those of their affiliated organizations, or those of the publisher, the editors and the reviewers. Any product that may be evaluated in this article, or claim that may be made by its manufacturer, is not guaranteed or endorsed by the publisher.

## References

[1] Cancer registry and statistics . National Cancer Center, Japan: Cancer Information Service (Accessed May 7, 2020).

[2] Hashiguchi Y Muro K Saito Y Ito Y Ajioka Y Hamaguchi T . Japanese Society for cancer of the colon and rectum (JSCCR) guidelines 2019 for the treatment of colorectal cancer. Int J Clin Oncol (2020) 25:1–42. doi: 10.1007/s10147-019-01485-z 31203527 PMC6946738

[3] Falcone A Ricci S Brunetti I Pfanner E Allegrini G Barbara C . Phase III trial of infusional fluorouracil, leucovorin, oxaliplatin, and irinotecan (FOLFOXIRI) compared with infusional fluorouracil, leucovorin, and irinotecan (FOLFIRI) as first-line treatment for metastatic colorectal cancer: the gruppo oncologico nord ovest. J Clin Oncol (2007) 25:1670–6. doi: 10.1200/JCO.2006.09.0928 17470860

[4] Cremolini C Loupakis F Antoniotti C Lupi C Sensi E Lonardi S . FOLFOXIRI plus bevacizumab versus FOLFIRI plus bevacizumab as first-line treatment of patients with metastatic colorectal cancer: updated overall survival and molecular subgroup analyses of the open-label, phase 3 TRIBE study. Lancet Oncol (2015) 16:1306–15. doi: 10.1016/S1470-2045(15)00122-9 26338525

[5] Heinemann V Modest DP von Weikersthal LF Decker T Kiani A Vehling-Kaiser U . Gender and tumor location as predictors for efficacy: influence on endpoints in first-line treatment with FOLFIRI in combination with cetuximab or bevacizumab in the AIO KRK 0306 (FIRE3) trial. J Clin Oncol (2014) 32:3600–6. doi: 10.1200/jco.2014.32.15_suppl.3600

[6] Brulé SY Jonker DJ Karapetis CS O’Callaghan CJ Moore MJ Wong R . Location of colon cancer (right-sided versus left-sided) as a prognostic factor and a predictor of benefit from cetuximab in NCIC CO. 17. Eur J Cancer (2015) 51:1405–14. doi: 10.1016/j.ejca.2015.03.015 25979833

[7] Arnold D Lueza B Douillard JY Peeters M Lenz HJ Venook A . Prognostic and predictive value of primary tumour side in patients with RAS wild-type metastatic colorectal cancer treated with chemotherapy and EGFR directed antibodies in six randomized trials. Ann Oncol (2017) 28:1713–29. doi: 10.1093/annonc/mdx175 PMC6246616 28407110

[8] Yoshino T Arnold D Taniguchi H Pentheroudakis G Yamazaki K Xu RH . Pan-Asian adapted ESMO consensus guidelines for the management of patients with metastatic colorectal cancer: a JSMO–ESMO initiative endorsed by CSCO, KACO, MOS, SSO and TOS. Ann Oncol (2018) 29:44–70. doi: 10.1093/annonc/mdx738 29155929

[9] Van Cutsem E Tabernero J Lakomy R Prenen H Prausová J Macarulla T . Addition of aflibercept to fluorouracil, leucovorin, and irinotecan improves survival in a phase III randomized trial in patients with metastatic colorectal cancer previously treated with an oxaliplatin-based regimen. J Clin Oncol (2012) 30:3499–506. doi: 10.1200/JCO.2012.42.8201 22949147

[10] Bennouna J Sastre J Arnold D Österlund P Greil R Van Cutsem E . Continuation of bevacizumab after first progression in metastatic colorectal cancer (ML18147): a randomised phase 3 trial. Lancet Oncol (2013) 14:29–37. doi: 10.1016/S1470-2045(12)70477-1 23168366

[11] Tabernero J Yoshino T Cohn AL Obermannova R Bodoky G Garcia-Carbonero R . Ramucirumab versus placebo in combination with second-line FOLFIRI in patients with metastatic colorectal carcinoma that progressed during or after first-line therapy with bevacizumab, oxaliplatin, and a fluoropyrimidine (RAISE): a randomised, double-blind, multicentre, phase 3 study. Lancet Oncol (2015) 16:499–508. doi: 10.1016/S1470-2045(15)70127-0 25877855

[12] Wirapati P Pomella V Vandenbosch B Kerr P Maiello E Jeffery GM . Velour trial biomarkers update: Impact of RAS, BRAF, and sidedness on aflibercept activity. J Clin Oncol (2017) 35:3538–46. doi: 10.1200/JCO.2017.35.15_suppl.3538

[13] Eisenhauer EA Therasse P Bogaerts J Schwartz LH Sargent D Ford R . New response evaluation criteria in solid tumours: revised RECIST guideline (version 1. 1). Eur J Cancer (2009) 45:228–47. doi: 10.1016/j.ejca.2008.10.026 19097774

[14] Greenwood M . The errors of sampling of the survivorship tables. Reports on Public Health and Medical Subjects. London: Her Majesty's Stationery Office, Vol. 33 (1926).

[15] Kaplan EL Meier P . Nonparametric estimation from incomplete observations. JASA (1958) 53:457–81. doi: 10.1080/01621459.1958.10501452

[16] Clopper CJ Pearson ES . The use of confidence or fiducial limits illustrated in the case of the binomial. Biometrika (1934) 26:404–13. doi: 10.1093/biomet/26.4.404

[17] Hofheinz RD Anchisi S Grünberger B Derigs HG Zahn MO Geffriaud-Ricouard C . Real-world evaluation of quality of life, effectiveness, and safety of aflibercept plus FOLFIRI in patients with metastatic colorectal cancer: the prospective QoLiTrap study. Cancers (2022) 14:3522. doi: 10.3390/cancers14143522 35884583 PMC9324206

[18] Kopetz S Hoff PM Morris JS Wolff RA Eng C Glover KY . Phase II trial of infusional fluorouracil, irinotecan, and bevacizumab for metastatic colorectal cancer: efficacy and circulating angiogenic biomarkers associated with therapeutic resistance. J Clin Oncol (2010) 28:453–9. doi: 10.1200/JCO.2009.24.8252 PMC2815707 20008624

[19] Nixon AB Sibley A Hatch AJ Liu Y Jiang C Mulkey F . Blood-based biomarkers in patients (pts) with metastatic colorectal cancer (mCRC) treated with FOLFOX or FOLFIRI plus bevacizumab (Bev), cetuximab (Cetux), or bev plus cetux: Results from CALGB 80405 (Alliance). J Clin Oncol (2016) 34:3597. doi: 10.1200/JCO.2016.34.15_suppl.3597 27601538

[20] Sims TN Gao B Chiron M Mancini P Dochy E Lowy I . Potential predictive and prognostic biomarkers identified in baseline plasma samples from the VELOUR trial. J Clin Oncol (2015) 33:638. doi: 10.1200/jco.2015.33.3_suppl.638

[21] Van Cutsem E Paccard C Chiron M Tabernero J . Impact of prior bevacizumab treatment on VEGF-a and PlGF levels and outcome following second-line aflibercept treatment: biomarker post hoc analysis of the VELOUR trial. Clin Cancer Res (2020) 26:717–25. doi: 10.1158/1078-0432.CCR-19-1985 31727675

